# *PIK3CA* and *PIK3R1* tumor mutational landscape in a pan-cancer patient cohort and its association with pathway activation and treatment efficacy

**DOI:** 10.1038/s41598-023-31593-w

**Published:** 2023-03-18

**Authors:** Zoé Tharin, Corentin Richard, Valentin Derangère, Alis Ilie, Laurent Arnould, François Ghiringhelli, Romain Boidot, Sylvain Ladoire

**Affiliations:** 1 Department of Medical Oncology, Centre Georges François Leclerc-UNICANCER, 1 Rue du Professeur Marion, 21000 Dijon, France; 2 Department of Pathology and Tumor Biology, Centre Georges François Leclerc, Dijon, France; 3 Platform of Transfer in Biological Oncology, Georges François Leclerc Cancer Center, Dijon, France; 4 University of Burgundy-Franche Comté, Dijon, France; 5 Centre de Recherche INSERM LNC-UMR1231, Dijon, France; 6 Genomic and Immunotherapy Medical Institute, Dijon University Hospital, Dijon, France; 7 ICMUB UMR CNRS 6302, Dijon, France

**Keywords:** Cancer, Breast cancer, Cancer epidemiology, Cancer genomics, Gynaecological cancer, Tumour biomarkers

## Abstract

There is little data concerning the implications of *PIK3CA* mutations outside of the known hotspots described in ER+/HER2− metastatic breast cancer (mBC). Similarly, *PIK3R1* mutations could also lead to activation of PI3K pathway, but are poorly described. We determined the incidence and type of all somatic *PIK3CA* and *PIK3R1* mutations by whole exome sequencing (WES) in a pan-cancer cohort of 1200 patients. Activation of the PI3K pathway was studied using phospho-AKT immunohistochemistry. Associations between *PIK3CA/PIK3R1* mutations and response to chemotherapy were studied in mBC cases. We found 141 patients (11.8%) with a *PIK3CA* and/or *PIK3R1* mutation across 20 different cancer types. The main cancer subtype was mBC (45.4%). Eighty-four mutations (62.2%) occurred in the three described hotspots; 51 mutations occurred outside of these hotspots. In total, 78.4% were considered activating or probably activating. Among *PIK3R1* mutations, 20% were loss of function mutations, leading to a constitutional activation of the pathway. Phospho-AKT quantification in tumor samples was in favor of activation of the PI3K pathway in the majority of mutated tumors, regardless of mutation type. In ER+/HER2− mBC, first line chemotherapy efficacy was similar for *PIK3CA-*mutated and *PIK3CA-*WT tumors, whereas in triple negative mBC, chemotherapy appeared to be more effective in *PIK3CA-*WT tumors. In this large, real-life pan-cancer patient cohort, our results indicate that *PIK3CA/PIK3R1* mutations are widely spread, and plead in favour of evaluating the efficacy of PI3K inhibitors outside of ER+/HER2− mBC and outside of hotspot mutations.

## Introduction

The phosphatidylinositol 3-kinase (PI3K)–AKT–mammalian target of rapamycin (mTOR) pathway is a key player in many cellular functions. Signaling through this pathway is essential to both physiological and malignant cellular processes, such as proliferation, adhesion, migration, invasion, metabolism, and survival^1–3^. In human cancer, up-regulation of this pathway is one of the most frequent events and is therefore an interesting therapeutic target^4,5^. Recently, new drugs targeting the PI3K-AKT-mTOR pathway have been developed, the latest approved drug being alpelisib, for the treatment of metastatic breast cancer (mBC) in patients with Endocrine Receptor positive (ER+), Human Epidermal Growth Factor 2 negative (HER2−) tumors and a hotspot mutation (HM) in Phosphatidylinositol-4,5-bisphosphate 3-kinase catalytic subunit alpha (*PIK3CA*)^6^.

Within the PI3K family, when it comes to regulation, proliferation and carcinogenesis, the most important PI3K proteins are those that belong to class IA. They are composed of a heterodimer protein made of a p110α catalytic subunit, encoded by *PIK3CA*, and a p85α regulatory subunit, encoded by phosphatidylinositol-4,5-bisphosphate 3-kinase regulatory subunit alpha (*PI3KR1*)^7^. Their role is to phosphorylate phosphatidylinositol-4,5-bisphosphate (PIP2) to produce Phosphatidylinositol-3,4,5-trisphosphate (PIP3) in order to activate the PI3K/AKT/mTOR pathway^8^.

To date, up to 160 mutations of the *PIK3CA* gene have been identified^9,10^. The most frequent mutations occur in 3 hotspots, and are the following: p.E542K and p.E545K in exon 10 (corresponding to the helical domain) and p.H1047 L/R in exon 21 (corresponding to the kinase domain), when starting to number the exons at the first transcribed exon. Hotspot mutations (HM) lead to constitutive activation of *PIK3CA* and oncogenic transformation into multiple cancers^11,12^. There is little data available concerning the description and implications of *PIK3CA* mutations in metastatic cancer apart from breast cancer (BC). In addition, it is unknown whether non-hotspot mutations (NHM) upregulate the PI3K/AKT/mTOR pathway and therefore whether p110α-selective drugs, such as alpelisib, would be effective in patients with NHM. In the absence of activating signals, p85α binds to, stabilizes and inhibits the catalytic activity of p110α. In the presence of growth factors or other signals, p85α activates the catalytic activity of p110α. When *PIK3R1* is mutated, the protein p85α cannot inhibit p110α’s catalytic activity, leading to constitutional activation of the pathway. There are no clinical trials evaluating the efficacy of p110α-selective drugs in this setting, and therefore it is unknown whether this drug class would be effective in a *PIK3R1* mutated population.

The cancer subtype where we have the most data concerning *PIK3CA* mutations is BC. Most analyses were carried out in early-stage BC, and have revealed that these mutations are more frequent in ER+ BC, elderly populations, low grade and small tumors^13^, all of these being factors associated with good prognosis. However, in a recent study, Chen et al*.* found that stage II and III breast cancers bearing a *PIK3CA* mutation, treated in a neoadjuvant setting with standard chemotherapy were associated with lower rates of pathological complete response (pCR), which could reflect chemoresistance in these tumors^12^. Older studies have proven that *PIK3CA* mutations were also associated with higher resistance rates to anti-HER2 treatment (for HER2 positive (HER2+) patients) and endocrine treatment (for ER+ and HER2− patients)^13–16^.

The aim of the present study was to describe the molecular epidemiology of *PIK3CA* and *PIK3R1* mutations in a large, real-life cohort of patients treated for cancer, whatever the cancer type, and who had tumor exome analysis performed by Next Generation Sequencing (NGS) at the *Centre George François Leclerc,* Dijon, France. Additional exploratory analyses concerning activation of the PIK3 pathway in tumor cells, and its link with treatment response were also performed in this cohort.

## Materials and methods

### Study population

We retrospectively screened the exomes of 1200 consecutive patients for *PIK3CA* and/or *PIK3R1* mutations. Patients were treated for cancer, whatever the cancer type or stage, at the *Centre George François Leclerc* between 02/09/2015 and 26/03/2019*.* Patients included in our analyses had a *PIK3CA* mutation, a *PIK3R1* mutation or both.

We evaluated the efficacy of chemotherapy, in terms of objective response rate (ORR) and progression-free survival (PFS) after the first line of metastatic chemotherapy, in the *PIK3CA*-mutated ER+/HER2− and triple negative breast cancer (TNBC) subgroups. We chose those subgroups because they were the largest, most homogeneous subgroups observed in our cohort. These two subgroups were compared to a *WT* population with the same histological characteristics. The *WT* population was extracted from the EXOMA (NCT02840604) database where whole exome sequencing by NGS was performed. This study was also carried out at the *Centre George François Leclerc*, Dijon, France. Patients for whom ORR or PFS to the first line of chemotherapy was unknown and patients who had only been treated by endocrine therapy, without any line of chemotherapy, were excluded from the analyses. The analyses were not performed in the *PIK3R1* population due to the low number of mutations observed.

### Ethical considerations

For patients included in the EXOMA trial (NCT02840604), all patients provided signed informed consent for the trial and genomic analysis. The trial protocol was approved by an institutional review committee (ethical committee and scientific review board from Georges François Leclerc Center) and the study was performed in accordance with the Declaration of Helsinki. The results are reported in compliance with the CONSORT checklist.

For patients not included in the EXOMA trial, only patients from whom informed consent was obtained and recorded in the medical chart were included in this retrospective study. WES analysis is performed as part of routine care in our center in order to find potentially targetable mutations. Before patients consented to WES of their tumoral tissue, they were informed by their oncologist. Germline testing was performed after genetic counselling by a clinical geneticist. The study was approved by the CNIL (French national commission for data privacy) and the local ethics committee, and was performed in accordance with the Helsinki Declaration and European legislation.

### DNA extraction

Formalin-Fixed Paraffin-Embedded (FFPE) tumors were analyzed by a pathologist to determine the tumor cell content. The samples were then macro-dissected to obtain a tumor cell content of at least 20%. Deoxyribonucleic acid (DNA) was extracted using the Maxwell-16 FFPE Plus LEV DNA purification kit (Promega) according to the manufacturer’s protocol. DNA quality was assessed by spectrophotometry with absorbance at 230, 260 and 280 nm. DNA was quantified using fluorometric assay with a Qubit device.

### Exome analysis

Two hundred nanograms (ng) of genomic DNA were fragmented with a Covaris device to obtain fragments of about 300 base pairs (bp). After that, DNA libraries were created from 200 ng DNA and captured by using SureSelect Human All Exon v6 kit (Agilent), following the manufacturer’s protocol. Paired-end (2 × 111 bases) sequencing was performed on a NextSeq500 device (Illumina). Obtained sequences were aligned and annotated with the human Hg19 genome, based on the SureSelect Human all Exon v6 manifest by using BWA and GATK algorithms. Variant Call Format (VCF) files were annotated with Variant Studio software (Illumina). Only variants with a missense mutation, a frameshift on canonical transcript, a read depth of 10x, a mutation allele frequency greater than 5% or a frequency in the general population less than 1% were conserved for the analysis. A gene was defined as altered if it had a least one of the variants described above. Mutations were classified as pathogenic, benign or of unknown significance by searching in different databases including the COSMIC, oncoKB, VARSOME and JAX databases. The database versions were those in use at the time of the analysis (January 2022). In the case of a variant of unknown significance, pathogenicity was determined by following the somatic variant guide according to the American College of Medical Genetics and Genomics (ACMG)/Association for Molecular Pathology (AMP) 2015 guidelines^17^. The 3 hotspots mutations in PIK3CA were p.E542K and p.E545K in exon 10 (corresponding to the helical domain) and p.H1047 L/R in exon 21 (corresponding to the kinase domain), when starting to number the exons at the first transcribed exon.

For each patient, we had either tumor DNA and their matched germline DNA or only tumor DNA. From patients with germline and tumor analyses, we were able to immediately detect the origin of mutations (tumor or germline). For patient with tumor DNA only, we checked the variant allele frequency and the tumor cell content. This allowed to assess whether the mutation signal was compatible with a tumor origin.

### Statistical analyses

Comparisons between the different groups of interest were performed using the Chi-2 or Fisher’s exact test for qualitative variables and the Mann–Whitney test for continuous variables, as appropriate. ORR to the first line of metastatic chemotherapy in ER+/HER 2– and TNBC according to *PIK3CA* mutational status was compared using Fisher's exact test. Survival curves for PFS and overall survival (OS) according to *PIK3CA* mutational status in the ER+/HER 2− and triple negative metastatic breast cancer settings, were estimated using the Kaplan–Meier method and compared with the log-rank test. All tests were two-sided and statistical significance was considered for p-values < 0.05. All statistical analyses were conducted using R software (v4.1.2; R Core Team 2021) and figures were drawn using GraphPad Prism.

### Immunohistochemistry and analysis

FFPE slides with a thickness of 4 µm were unwaxed and stained using a PT link (Agilent) and an Autostainer 48 (Agilent). To summarize, slides were unwaxed using pH9 buffer for 20 min at 95 °C. After cooling, slides were washed in buffer (Agilent) twice for 5 min. Peroxydase blocking was performed using S2023 solution (Agilent) for 5 min. Phospho-AKT (1/100, clone D9E, CST) antibody was then applied for 60 min at room temperature (RT). Labelled polymers (SM8002—Agilent) were added for 20 min at RT after repeating the washing process twice. DAB (SM803) was then added to the specimen for 10 min. After new washing steps, slides were finally incubated with hematoxylin (Enzo) for 20 min and permanently mounted using a Leica automated coverslipper. All these IHC analyses were performed on the tumor sample on which the exome analyses were carried out for the determination of the presence of the *PIK3CA* and *PIK3R1* mutations. Positive controls used for IHC staining were breast tumors harbouring one of the PIK3CA hotspot activating mutations (E545K and H1047R, evaluated by WES), while negative controls were breast tumors with no mutations on the PI3KCA/AKT/mTOR pathway (evaluated by WES).

Slides were then digitalized with a Nanozoomer HT2.0 slide scanner (Hamamatsu) at 20X and analyzed with QuPath software (v2)^18^. At least five representative tumor areas per slide were marked by an expert pathologist and staining intensity was evaluated with a H-score and Allred score strategy. Quantification of p-AKT staining using the H-score strategy was restricted to epithelial cancer cells. This staining was specific, and no background was detected, even in necrotic or acellular areas. To calculate the H-score, the software needed to detect cells first with a haematoxylin nuclei detection algorithm. Then, within these detected cells, chromogen intensity was calculated.

While the H-score, spanning from 0 to 300^19^, considers a ratio of each staining intensity (1+, 2+, 3+) on total detected cells with weighting (1 for 1+, 2 for 2+ and 3 for 3+), the Allred score, spanning from to 0 to 8^20^, is the sum of a proportion score (from 0 to 5) and an intensity score (from 0 to 3). The results displayed here are the mean of the areas marked by the pathologist. Thresholds were set using negative and positive controls. The QuPath script used for this work is available on request.

## Results

### Patient characteristics

Among the 1200 patients who underwent tumor exome analysis, we found a total of 141 patients (representing 11.75% of the screened population) with *PIK3CA* or *PIK3R1* somatic mutation (Supplemental Fig. 1). Female gender was predominant, with 118 women (83.7%) and 23 men (16.3%). The mean age at initial cancer diagnosis was 59.2 years for men, and 54.3 years for women. Performance status at time of exome analysis was 0 or 1 in 70.2% of patients. The main histology was adenocarcinoma (113 patients). One hundred and twenty-nine (91.5%) patients had metastatic disease, 5 patients had early-stage cancer, and the status was unknown for 7 patients. One hundred and twenty-eight patients (90.7%) had a *PIK3CA* mutation (of whom 5 (3.5%) had a double *PIK3CA* mutation), 11 patients (7.8%) had a *PIK3R1* mutation (of whom 2 (1.4%) had a double *PIK3R1* mutation), and finally, 2 patients (1.4%) had both a *PIK3CA* and a *PIK3R1* mutation (Table 1).

**Table 1:** Patients’ characteristics.

Variable	Category	Total N = 141	
Gender	Male	23 (16.31%)	
	Female	118 (83.69%)	
Mean age at initial diagnoses	Male	59.24 (39–73)	
	Female	54.36 (20–81)	
Performance status at initial diagnoses	0–1	99 (70.21%)	
	≥ 2	5 (3.55%)	
	Unknown	37 (26.24%)	
Histology	Adenocarcinoma	113 (80.14%)	
	Squamous cell carcinoma	15 (10.64%)	
	Sarcoma	2 (1.42%)	
	Urothelial carcinoma	2 (1.42%)	
	Melanoma	1 (0.71%)	
	Follicular thyroid cancer	1 (0.71%)	
	Glioblastoma	1 (0.71%)	
	Unknown	6 (4.26%)	
Metastatic or locally advanced disease	Yes	129 (91.49%)	
	No	5 (3.55%)	
	Unknown	7 (4.96%)	
Time of metastatic disease	Synchronous	44 (31.21%)	
	Metachronous	84 (59.57%)	
	Not concerned	5 (3.55%)	
	Unknown	8 (5.67%)	
Mutated gene	*PIK3CA*	128 (90.78%)	
	*PIK3R1*	11 (7.80%)	
	*PIK3CA* + *PIK3R1*	2 (1.42%)	

**Figure 1:**
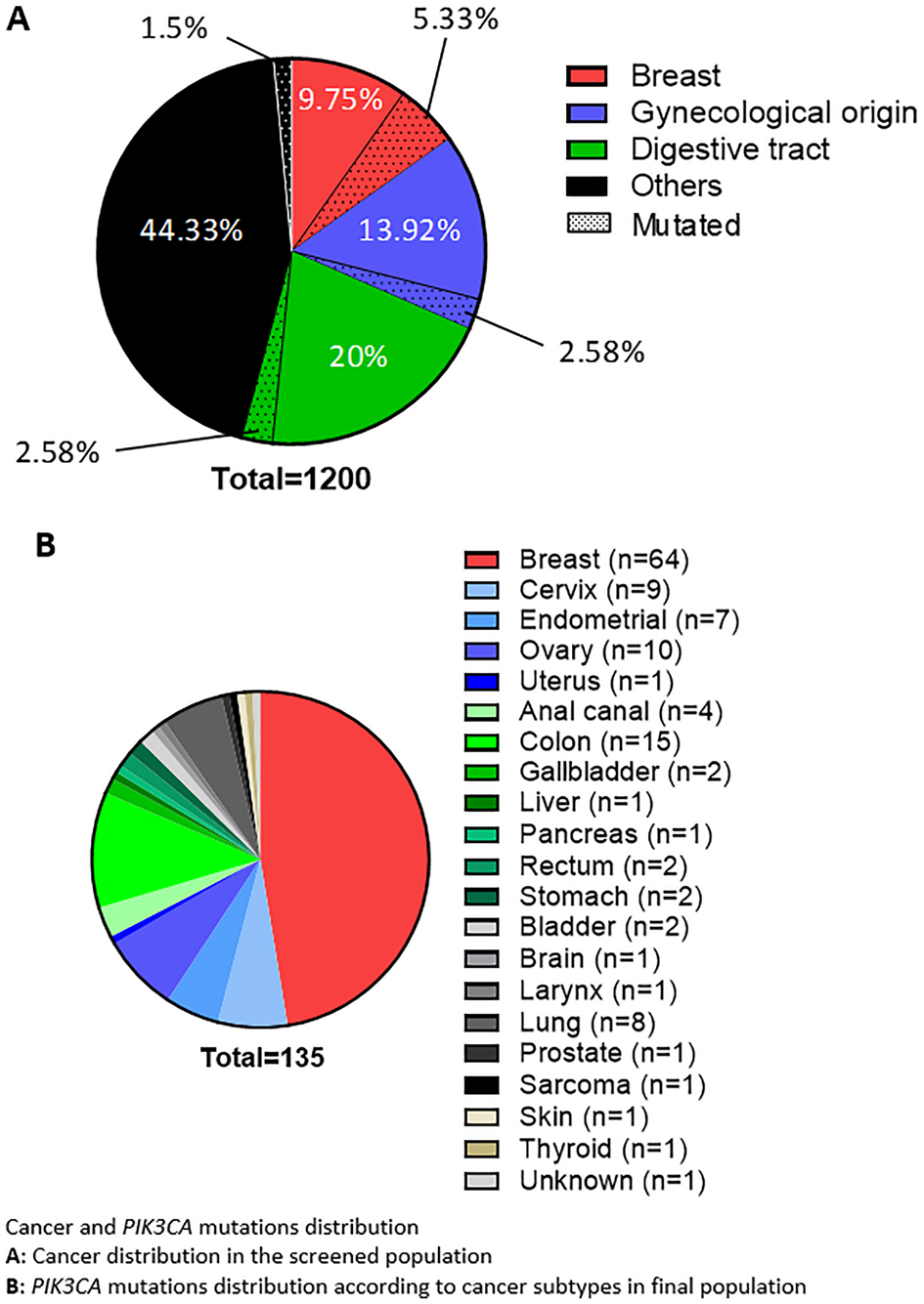
Cancer and *PIK3CA* mutations distribution (**A**) Cancer distribution in the screened population. (**B**) *PIK3CA* mutations distribution according to cancer subtypes in final population.

The cancer distribution was very different in the initial, total screened population and in the final analysis population. In the screened population, there was 15.1% breast cancers, 16.5% gynecological cancers, 22.6% digestive tract cancers and 45.8% other cancer types (Fig. 1A). In the final population analysed, there was an enrichment of breast cancers, representing 59 patients (45.4%) and 64 *PIK3CA* mutations (47.4%). Digestive tract cancers and gynecological cancers were equally represented with 27 patients (20.8%) each and 27 *PIK3CA* mutations (20%). Other cancer types accounted for 28 patients (21.5%) and 28 *PIK3CA* mutations (20.7%) (Fig. 1B). A long tail plot of the distribution of PIK3CA and PIK3R1 mutations across disease groups is presented in Supplemental Fig. 2 (Prevalence: Supplemental Fig. 2A,B, and number: Supplemental Fig. 2C,D). In light of these results (Supplemental Table 1), we decided to focus on breast, gynecological and digestive tract cancers, as the other locations were poorly represented. We also decided to pool the different gynecological and digestive tract cancers in order to have sufficient numbers for robust analysis.

### Mutational landscape of *PIK3CA*

**Figure 2:**
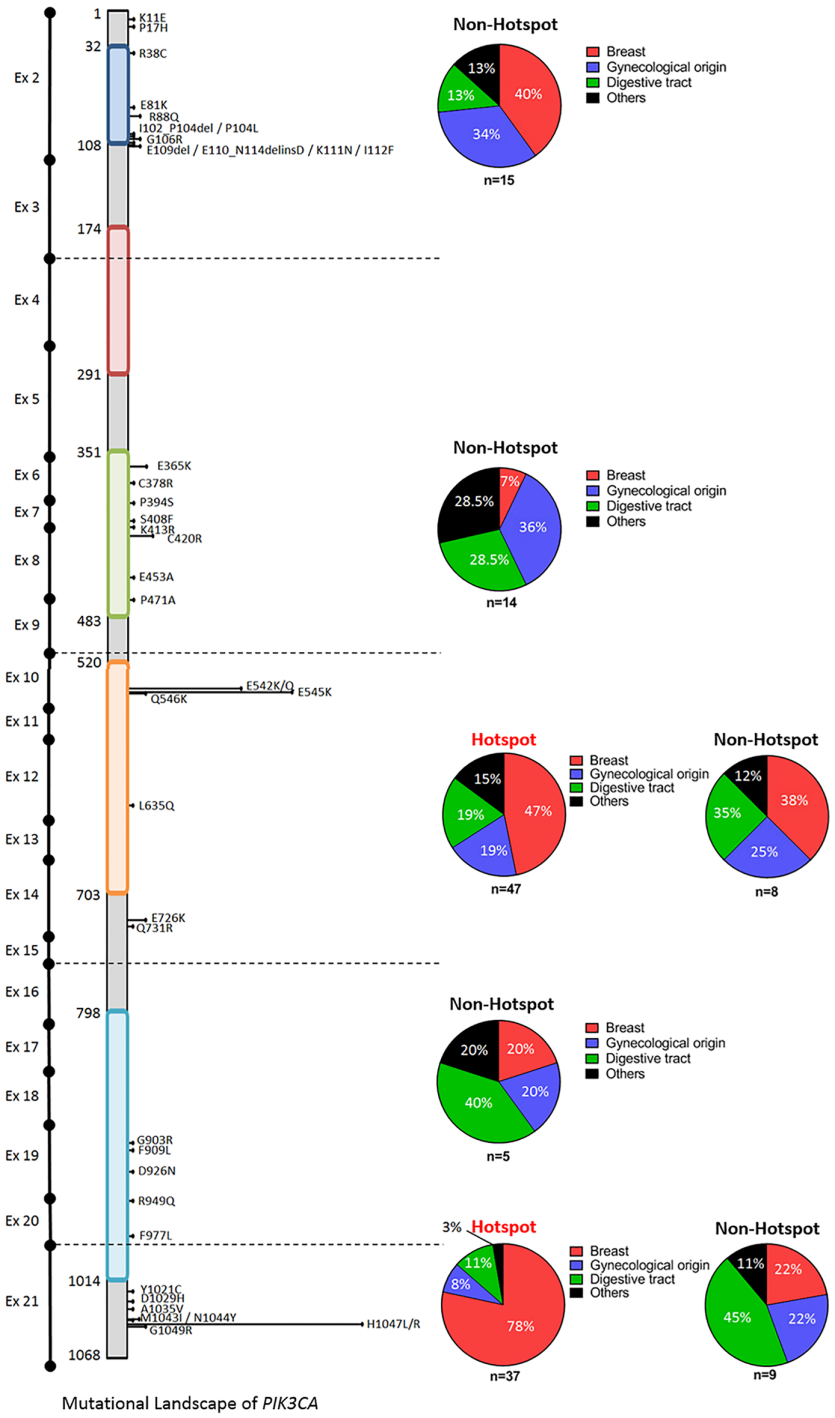
Mutational landscape of *PIK3CA*.

We analyzed a final total of 135 different *PIK3CA* mutations (123 single mutations + 5 double mutations + 2 mutations associated with *PIK3R1* mutations; see Supplemental Fig. 1). These 135 *PIK3CA* mutations were divided between 38 different mutations. Eighty-four mutations (62.2%) occurred in the 3 known hotspots: 47 mutations occurred in exon 10 for the mutations p.E542K and p.E545K, and 37 mutations occurred in exon 21 for the mutations p.H1047 L/R. Therefore, 51 mutations were non-hot spot mutations (NHM) and were divided into 35 different mutations across exons 2, 6, 7, 8, 10, 12, 14, 19, 20 and 21, when numbering from the first transcribed exon. The 3 main tumor locations associated with *PIK3CA* mutations were breast, digestive tract and gynecological cancers. Breast and gynecological cancers were the most frequent tumor types for NHM of exon 2, with respectively 40% and 34% of 15 mutations. Gynecological and digestive tract cancers were the most frequent tumor types for the NHM of exons 6 to 8, with respectively 36% and 28.5% of 14 mutations. The majority of exon 10 hot spot mutations (HM) were associated with breast cancer, with 47% of 47 mutations; gynecological and digestive tract cancers each represented 19% of these mutations. NHM of exons 10, 12 and 14 were relatively evenly distributed between breast, digestive tract and gynecological cancers, with respectively 38%, 35% and 25% of 8 mutations. The last HM, i.e. mutation p.H1047 L/R of exon 21, was highly associated with breast cancer, with 78% of the 37 mutations found in patients with breast cancer. NHM of exon 21 were more frequently found in patients with digestive tract cancers: 47% of 9 mutations (Fig. 2). No co-mutation of AKT and TOR was found in our series.

### Mutational landscape of *PIK3R1*

**Figure 3:**
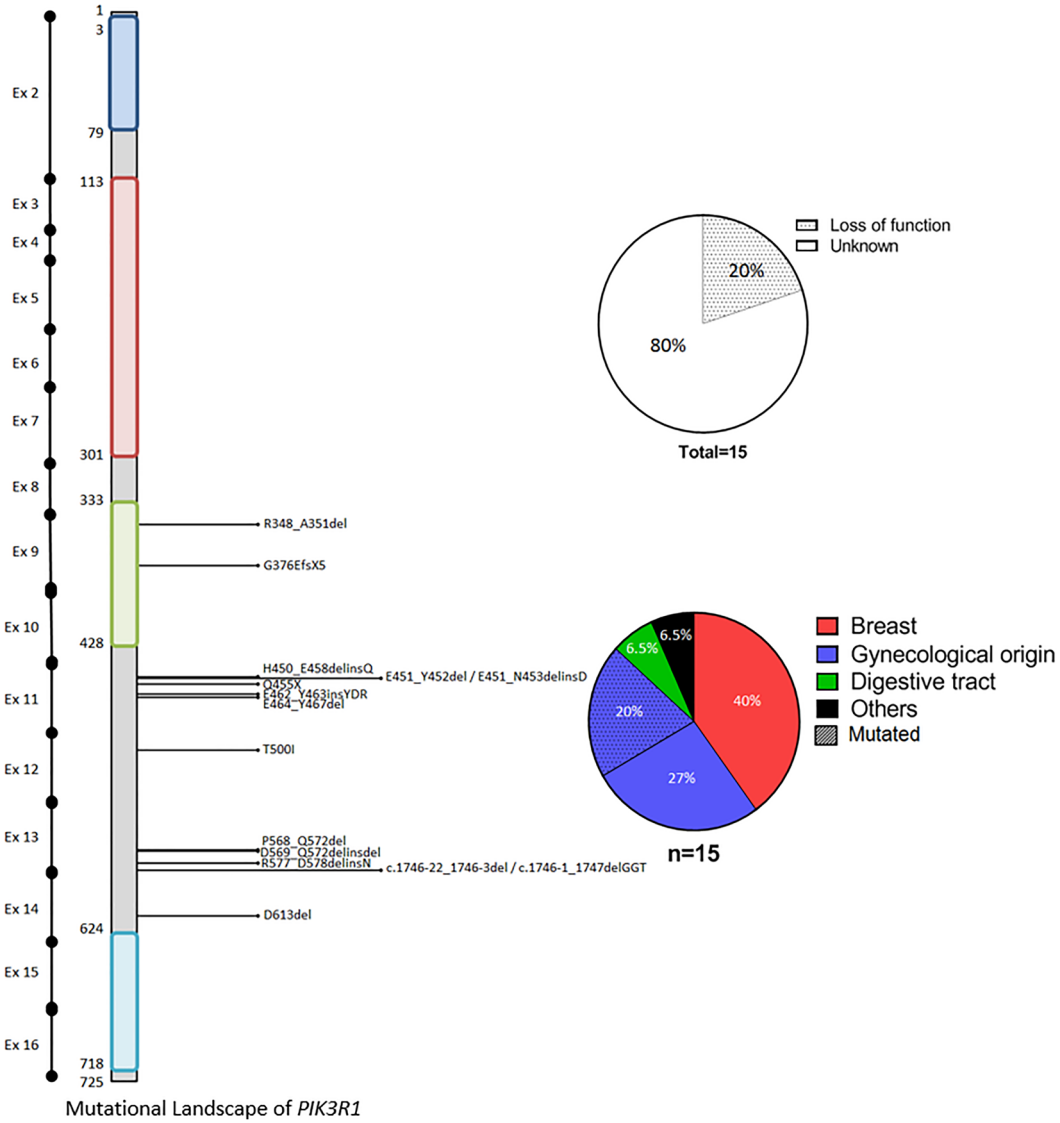
Mutational landscape of *PIK3R1*.

*PIK3R1* mutations were less frequent, with a total of 15 mutations found in our cohort. The most affected exons were exons 11 and 13, with respectively 6 and 5 different mutations. Twenty percent of all mutations were loss of function mutations, leading to constitutional activation of the pathway, and they were all found in patients with gynecological cancers. The effect of the *PIK3R1* mutation was unknown in the remaining 80% of cases (Fig. 3).

### Distribution of tumor type according to the different *PIK3CA* mutations

**Figure 4:**
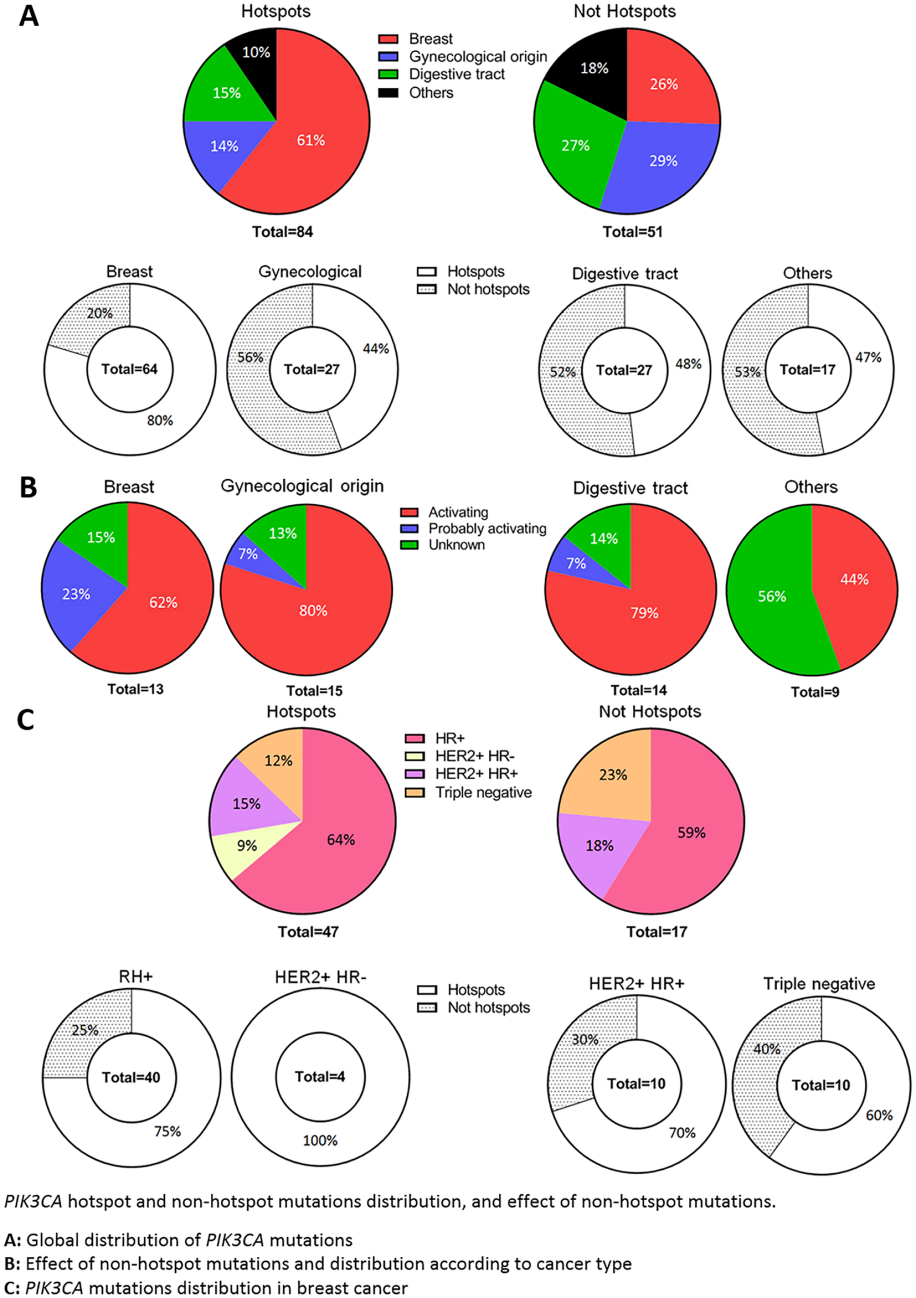
*PIK3CA* hotspot and non-hotspot mutations distribution, and effect of non-hotspot mutations. (**A**) Global distribution of *PIK3CA* mutations. (**B**) Effect of non-hotspot mutations and distribution according to cancer type. (**C**) PIK3CA mutations distribution in breast cancer.

Sixty-one percent of *PIK3CA* HM were accounted for by breast cancers, whereas 80% of *PIK3CA* mutations in patients with breast cancer were HM. For gynecological and digestive tract cancers, the distribution was quite homogeneous between HM and NHM. For instance, gynecological cancers represented 14% of HM and 27% of NHM. When looking at the distribution within the gynecological cancers, 44% of mutations occurred in hotspots and 56% outside of hotspots. The distribution for digestive tract cancers was similar (Fig. 4A).

For breast, gynecological and digestive tract cancers, NHM *PIK3CA* mutations were presumed activating or probably activating (and therefore leading to constitutional activation of the pathway) in 78.4% of cases. We observed the highest proportion of unknown effect (56% of cases) in the other cancer subtypes of our cohort (melanoma, sarcoma, glioblastoma, etc) (Fig. 4B).

Looking more closely at breast cancers and their different histological subtypes, we observed that ER+ and HER2- tumors were the most frequently represented, regardless of the mutation site. Indeed, 64% of HM and 59% of NHM associated with breast cancer were found in this ER+ and HER2− histological subtype. Nevertheless, ER+/HER2+ and triple negative tumors were also well represented with respectively 15% and 12% of HM; and 18% and 23% of NHM. The ER negative (ER−) and HER2+ subtype was poorly represented with only 9% of HM, and no NHM (Fig. 4C).

### Phospho-AKT tumor status by immunohistochemistry

**Figure 5:**
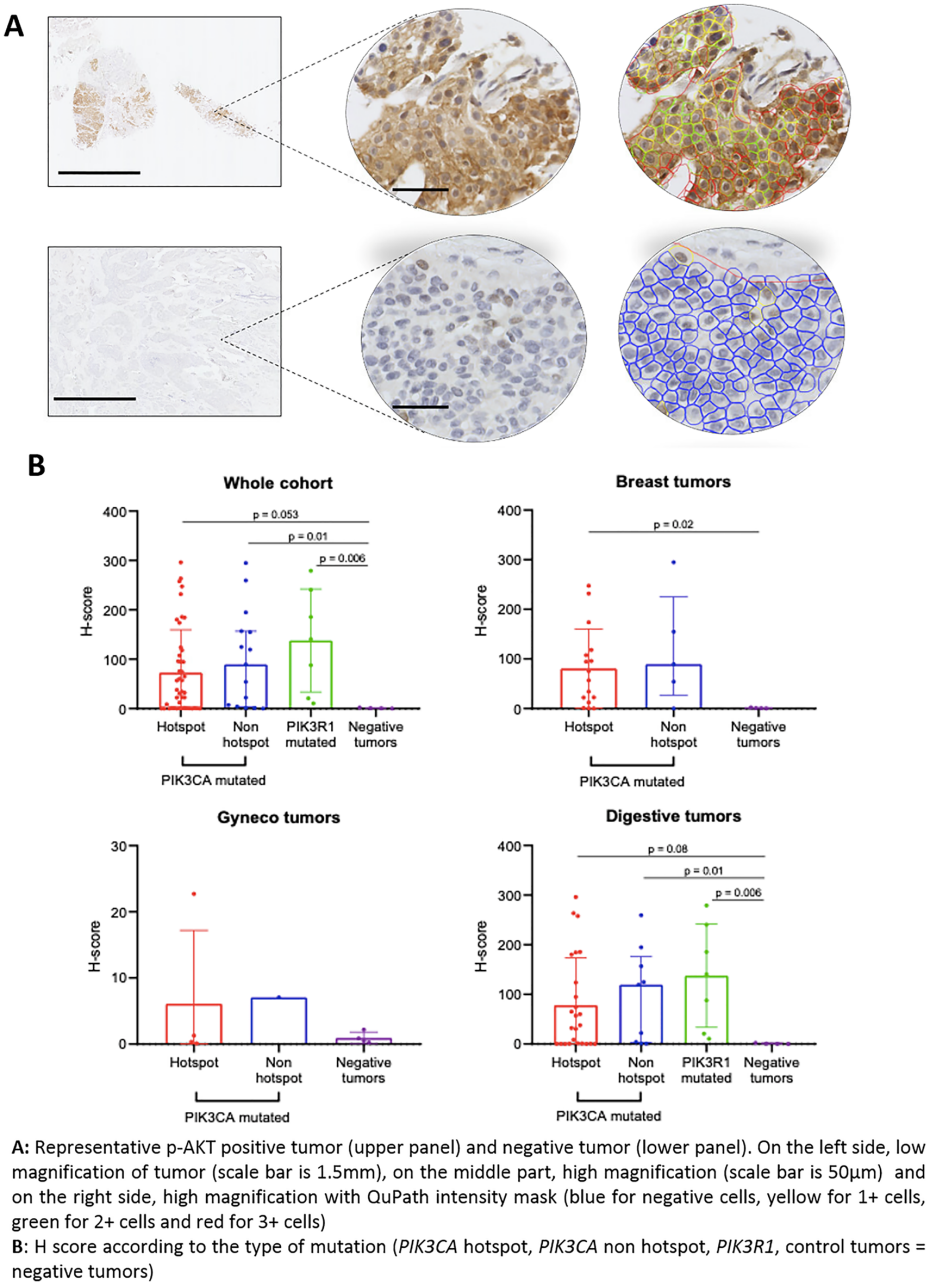
(**A**) Representative p-AKT positive tumor (upper panel) and negative tumor (lower panel). On the left side, low magnification of tumor (scale bar is 1.5 mm), on the middle part, high magnification (scale bar is 50 µm) and on the right side, high magnification with QuPath intensity mask (blue for negative cells, yellow for 1+ cells, green for 2+ cells and red for 3+ cells). (**B**) H score according to the type of mutation (*PIK3CA* hotspot, *PIK3CA* non hotspot, *PIK3R1*, control tumors = negative tumors) .

As we found both HM and NHM (with various degrees of pathogenicity) in our cohort, we then sought to evaluate the activation of the PI3K pathway using immunohistochemistry, by studying the presence of phospho-AKT in the tumor cells (Fig. 5A) of the tumor sample on which the exome analyses were carried out for the determination of the presence of *PIK3CA* and *PIK3R1* mutations. For this exploratory analysis, we focused only on breast, digestive and gynecological cancers. Seventy-one tumor samples were available. There were 64 *PIK3CA* mutations (47 HM, 17 NHM) and 7 *PIK3R1* mutations. The *PIK3CA* NHM mutations were activating or probably activating in 15 cases, while the effect of the mutation was unknown in two cases. The effect of the *PIK3R1* mutations was unknown in all cases. A H score was established in the 71 tumor samples bearing the mutations described above, and in five control tumors (with no somatic mutations in genes involved in the PIK3/AKT/mTOR pathway) (Fig. 5A). The cancer type, mutations and H scores are summarized in Supplemental Table 2.

The H score was close to zero for the control tumors. For tumors bearing a *PIK3CA* mutation, the H score ranged from 0.0145 to 296.1767. H scores from *PIK3CA* mutated tumors were significantly higher than control tumors, both for HM and NHM cases (Fig. 5B). There was no significant difference in H score between HM and NHM cases. Concerning tumors bearing a *PIK3R1* mutation, H score ranged from 10.3888 to 279.03, and was significantly higher than control tumors (Fig. 5B), but non significantly different when compared to H scores from HM and NHM *PIK3CA* mutated tumors (despite all *PIK3R1* mutations being classified “unknown” in terms of pathogenicity). The median values of the H scores with inter quartile ranges (IQR) of all groups are detailed in Supplemental Table 3.

Although exploratory, these results plead in favor of activation of PI3K pathway in the majority of mutated tumors. Similar trends were observed in breast, digestive, and gynecological cancers.

### Prognostic and predictive value of *PIK3CA* mutations in breast cancer

As our series included many cases of metastatic breast cancer, we also carried out exploratory analysis of the association between *PIK3CA* mutations and response to different types of anti-cancer treatments, such as chemotherapy and pharmacological agents targeting the PI3K-AKT-mTOR pathway.

The ER+/HER2− *PIK3CA*-mutated group included 31 patients (28 patients with a *PIK3CA* HM, 3 patients with a *PIK3CA* NHM). These patients were compared to a group of 54 patients (included in the 1200 initially screened patients, with available tumor exome) with ER+/HER2− tumors, but with *PIK3CA-WT* status. The triple negative *PIK3CA* group included only six patients (5 patients with a PIK3CA HM, 1 patient with a PIK3CA NHM), and was compared to a group of 16 patients with triple negative characteristics and *PIK3CA-WT* status. Due to the low number of NHM, and considering our previous results in favor of activation of the PI3K pathway also in tumors with NHM, we decided to pool them with the HM for the purposes of this analysis.

As *P53* is also frequently mutated in MBC, we explored whether *P53* mutations were associated with *PIK3CA* mutations. We did not find any statistically significant association between these two mutations. The proportion of *P53* mutated tumors was not statistically different in mutated *PIK3CA* (29.5%) compared to *PIK3CA* WT (30.9%) MBC (Chi-2 test p = 0.97). Similarly, co-mutations of PIK3CA and P53, had no impact on PFS (first line of chemotherapy) compared with PIK3CA mutation only (data not shown).

### Association between *PIK3CA* mutation and response to first line chemotherapy

In the ER+/HER2− cohort, the ORR to the first line of chemotherapy was similar between the *PIK3CA* mutated group and the *WT* group, with respectively 12.9% versus 13% complete responses (CR), 64.5% versus 53.7% partial responses (PR), 3.2% versus 14.8% stable disease (SD) and 19.3% versus 18.5% progressive diseases (PD). There was no significant difference between groups in terms of PFS (1st line chemotherapy) or OS (Supplemental Fig. 3A–C).

For the triple negative cohort, we observed significant differences in terms of ORR to the first line of metastatic chemotherapy and in terms of PFS when comparing the *PIK3CA* mutated group to the *WT* group. Regarding ORR to the first line of metastatic chemotherapy, the results were as follows: no CR in the *PIK3CA* mutated group versus 25% in the *WT* group, 50% PR in both groups, and 50% PD in the *PIK3CA* mutated group versus 25% in the *WT* group. PFS was 4.5 months for the *PIK3CA* mutated group versus 12.7 months for the *WT* group and these results were statistically significant with p = 0.01. This was consistent when regarding OS, which was 11.9 months in the mutated group versus 33.1 months in the *WT* group. However, these results did not reach statistical significance (p = 0.16) (Supplemental Fig. 4A–C).

### Association between *PIK3CA* mutation and response to PI3K-AKT-mTOR targeted therapies

In our cohort, 35 patients received pharmacological targeting of the PIK3–AKT–mTOR pathway. Thirty-two received everolimus, two received alpelisib and one received taselisib. These treatments were used quite late in the disease course, on average as the fourth line of treatment. When looking at the whole cohort, PFS was 3.5 months. For the *PIK3CA* NHM group, PFS reached 6.5 months, versus 3.4 months for the HM group. However, this difference was not significant (p = 0.33) and the only CR was observed in the HM group (in a mTNBC patient). PFS was not correlated with H-score, although the only CR was found in the high H-score group (defined as a score above the median value) (Supplemental Fig. 5).

## Discussion

Characterizing molecular mutations has become a new goal in oncology in recent years, since it makes it possible to use targeted therapies, which can offer disease control and often, better tolerance than standard chemotherapy. Here, we describe the different *PIK3CA* and *PIK3R1* mutations observed in a real-life cohort of 1200 cancer patients. We found a total of 135 somatic *PIK3CA* mutations (among which 38 different mutations, located in various exomes) and 15 *PIK3R1* mutations (among which 20% were considered pathogenic because loss-of-function), across 20 different cancer types. The results presented here are original because they describe the prevalence of PIK3CA mutations, not only in hotspots, but also outside mutational hotspots, in a large, pan-cancer cohort representative of the real-life clinical activity of a cancer center. Moreover, in the same series, we studied PIK3R1 mutations, for which published descriptive data are scarce. Additionally, our exploratory results on the functional consequences of non-hotspot mutations and PIK3R1 mutations could suggest the possible therapeutic value of targeting the PI3K pathway.

As expected, we confirmed in our study that *PIK3CA* mutations are widely spread in different cancer locations. Our results are consistent with what is described in literature, with a *PIK3CA* mutation being detected in over 10% of all cancer patients^21^. Eighty-four (62.2%) of the *PIK3CA* mutations were found in the 3 hotspots (p.E542K and p.E545K in exon 10; p.H1047 in exon 21). The remaining 51 (37.8%) mutations were distributed across 35 NHM.

Not surprisingly, breast cancer was largely represented in our cohort of *PIK3CA* mutations. Indeed, 58 patients (45.4%) with a *PIK3CA* mutation had breast cancer. Proportionally, this accounts for 32% of the 181 patients initially screened with breast cancer, whatever the histological subtype. These results are consistent with previous observations in the literature^13,22^. Eighty percent of mutations observed in patients with breast cancer were harbored in the three known hotspots. There are fewer data in the literature concerning *PIK3CA* mutations in gynecological or digestive cancers. We found that within these populations, the distribution of HM and NHM was relatively even. These results should be interpreted, bearing in mind that even by pooling all the gynecological and digestive tract cancer subtypes, our groups were rather small, with 27 mutations each.

The field of *PIK3CA* mutations has recently garnered increasing interest, with the clinical results of the SOLAR1 trial showing an improvement in PFS with alpelisib (an α-selective PI3K inhibitor) in patients whose tumor presented a somatic *PIK3CA* mutation in one of the three mutational hotspots^6^.

Our study confirms the very wide variety of *PIK3CA* mutations identified in cancer. The most frequent are HM single amino acid substitutions in the helical (E545K and E542K in exon 10), or kinase (H1047R in exon 21) domains^23^. Numerous mutations outside of these 3 hotspots were identified. These NHM are probably underestimated in mBC, but also in cancer in general, since most clinical studies focus on the 3 HM. Although less frequent, the majority of these NHM seem to lead to partial activation of PI3K in cellular models^24^. In cancer patients, the functional consequences of these NHM are unclear, but seem to converge towards a structurally open PI3K complex, increasing membrane binding and kinase activity owing to less inhibition of p110α by p85α^25^. This is how some rare *PIK3CA* mutations have been associated with endocrine resistance in ER+/HER2− mBC^26^. Our analysis of AKT phosphorylation in tumor cells from HM and NHM tumor samples support this hypothesis, by showing in particular similar levels of p-AKT in the HM and NHM groups, and significantly higher than in control tumors devoid of PI3K pathway mutation. However, these data can only be considered as exploratory, since there is currently no standardized method of assessing p-AKT, and the level of AKT phosphorylation beyond which one can consider the PI3K-AKT-mTOR pathway to be activated is unknown.

In mBC, most *PIK3CA* mutations occur in ER+/HER2− tumors, but interestingly, PI3K/AKT/PTEN pathway alteration is also described in 25–40% of patients with mTNBC^27^. *PIK3CA* mutations occur in around 10% of early TNBC, and their frequency increases in metastatic forms, reflecting a subset of originally ER+ breast cancer that relapse, losing their ER expression, and thus becoming “secondary” TNBC. In our cohort, we found that 13.8% of all TNBC had *PIK3CA* mutation. The majority of these cases (60%) harboured a HM, and in our small series of cases, we report the clinical observation of one of these patients presenting partial response to alpelisib, thus suggesting that some mTNBC could be candidates for an α-selective PI3K inhibitor. Numerous clinical trials are ongoing to test PI3K or AKT inhibitors, either alone or in combination, plus chemo- and/or immunotherapy^27^.

*PIK3R1* mutations were less frequent, with only 15 mutations observed in total, which were all different. Since it is rarer than *PIK3CA*, the *PIK3R1* mutational landscape is less well described in the literature. According to Sjöblom et al. and Philp et al., *PIK3R1* mutations are more commonly found in ovary, colon and breast cancers^28,29^. According to Cheung et al., *PIK3R1* mutations are particularly prevalent in endometrial cancer, glioma and colon cancer^30^. These conflicting results reflect the sparse knowledge we have about *PIK3R1* mutations. In our cohort, breast and endometrial cancers each harbored 40% of the PIK3R1 mutations. In the preclinical study by d’Ambrosio et al., cell lines with a PIK*3R1*^W624^^R^ mutation were sensitive to the pan-class-I PI3K inhibitor buparlisib, and to the p110α-specific inhibitor alpelisib, when compared to control cell lines^31^. The same conclusions were drawn from other preclinical studies, as in the work of Thorpe et al.^5^*.* These results are encouraging regarding the potential efficacy of PIK3 inhibitors in patients with a *PIK3R1* mutation, and warrant confirmation in further clinical trials.

There are contradictory results in the various published studies concerning the efficacy of chemotherapy in the *PIK3CA*-mutated BC population. According to Chen et al*.* and Yuan et al*.,* in early stage BC, *PIK3CA* mutations were associated with lower pCR rates^12,32^. These results could suggest that a *PIK3CA* mutation induces a certain level of chemoresistance in breast cancer*.* This has been suggested in other tumor locations, such as colorectal cancer, where Wang et al*.* evaluated resistance to first line chemotherapy in a *PIK3CA* mutated population compared to a *WT* population. The resistance rates were higher in the *PIK3CA* mutated group than in the *WT* group, with recurrence rates of respectively 71.4% and 44.5%^33^. In the setting of BC, again in the setting of early stage cancer, Cornelia et al*.* found that there was no statistically significant difference in terms of pCR to neoadjuvant chemotherapy between *PIK3CA*-mutated and *WT* groups^34^. In the metastatic setting, our findings point in the same direction for the ER+/HER2− histological subgroup. Indeed, we found similar ORR to the first line of chemotherapy in the *PIK3CA* mutated and *WT* groups, and no significant difference was observed in PFS between groups. These results are very exploratory, considering the small group of heterogeneous patients that we analyzed, and the findings are in contradiction with the largest reported cohort of patients, in which those with somatic *PIK3CA* HM had poorer response to chemotherapy and poorer survival^11^. In the same publication, the authors also reported the impact of *PIK3CA* mutation in mTNBC, and found improved prognosis in *PIK3CA*-mutated cases^11^. These results are also in contradiction with ours, but here again, our results must be interpreted with caution, given the small number of cases (and the unknown proportions of “real” TNBC and “secondary TNBC” in each group).

As described, the principal limitations of our study, in particular for the evaluation of ORR and PFS to first line chemotherapy, are its retrospective nature, the single-centre recruitment and the small subgroups of patients.

In conclusion, *PIK3CA* mutations are widely spread across all cancer types and represent a vast entity of different mutations. To date, HM have been of therapeutic interest only in ER+/HER2− BC. However, in our cohort, 39% of HM occurred outside of the BC spectrum, and could therefore become an interesting target in many other cancers. Similarly, NHM mutations are frequent and represented 37.8% of the *PIK3CA* mutations of our cohort. In our analysis, the vast majority of these NHM were considered activating or probably activating, and could lead to upregulation of the PI3K/AKT/mTOR pathway. *PIK3R1* mutations are rarer, but are far from being insignificant, and patients bearing such mutations may also benefit in some cases from *PIK3CA* targeted therapies. It would be interesting to evaluate the efficacy of *PI3KCA* inhibitors, such as alpelisib, in clinical trials in these three different settings.

## Supplementary Information

Supplementary Figure 1.

Supplementary Figure 2.

Supplementary Figure 3.

Supplementary Figure 4.

Supplementary Figure 5.

Supplementary Table 1.

Supplementary Table 2.

Supplementary Table 3.

Supplementary Legends.

## Data Availability

The datasets used and/or analysed during the current study are available from the corresponding author on reasonable request.
