# Supplementary material for: PIK3CA and PIK3R1 tumor mutational landscape in a pan-cancer patient cohort and its association with pathway activation and treatment efficacy
Source: Sci Rep. 2023 Mar 18;13:4467. doi: 10.1038/s41598-023-31593-w (PMC10024711; doi:10.1038/s41598-023-31593-w)
Supplement: Supplementary file 6 — Supplementary Table 1. [file 41598_2023_31593_MOESM6_ESM.docx]

| **Cancer type** | **N = 141** | |  | **Mutations** | |
| --- | --- | --- | --- | --- | --- |
|  |  |  |  | ***PIK3CA* N=135** | ***PIK3R1* N = 15** |
| **Breast** | **64 (45.39%)** | |  | **64 (47.40%)** | **6 (40%)** |
| **Gynecological** | **31 (21.99%)** | |  | **27 (20%)** | **7 (46.67%)** |
|  | Cervix | 8 |  | 9 | 0 |
|  | Uterus | 1 |  | 1 | 1 |
|  | Endometrium | 11 |  | 7 | 5 |
|  | Ovary | 11 |  | 10 | 1 |
| **Digestive tract** | **28 (19.86%)** | |  | **27 (20)** | **1 (6.67%)** |
|  | Anal canal | 5 |  | 4 | 1 |
|  | Colon | 15 |  | 15 | 0 |
|  | Stomach | 2 |  | 2 | 0 |
|  | Liver | 1 |  | 1 | 0 |
|  | Pancreas | 1 |  | 1 | 0 |
|  | Rectum | 2 |  | 2 | 0 |
|  | Gallbladder | 2 |  | 2 | 0 |
| **Other** | **18 (12.77%)** | |  | **17 (12.6%)** | **1 (6.67%)** |
|  | Lung | 8 |  | 8 | 0 |
|  | Brain | 1 |  | 1 | 0 |
|  | Larynx | 1 |  | 1 | 0 |
|  | Skin | 1 |  | 1 | 0 |
|  | Prostate | 1 |  | 1 | 0 |
|  | Sarcoma | 1 |  | 1 | 0 |
|  | Thyroid | 1 |  | 1 | 0 |
|  | Bladder | 2 |  | 2 | 0 |
|  | Inconnu | 2 |  | 1 | 1 |

**Supplemental Table 1**: PIK3CA and PIK3R1 mutations according to cancer types
