# Supplementary material for: PIK3CA and PIK3R1 tumor mutational landscape in a pan-cancer patient cohort and its association with pathway activation and treatment efficacy
Source: Sci Rep. 2023 Mar 18;13:4467. doi: 10.1038/s41598-023-31593-w (PMC10024711; doi:10.1038/s41598-023-31593-w)
Supplement: Supplementary file 7 — Supplementary Table 2. [file 41598_2023_31593_MOESM7_ESM.docx]

**Supplemental Table 2:**

Cancer type, mutations and H scores in tumor samples in which phospho-AKT status was evaluated by immunohistochemistry.

| **Organ** | **Gene** | **Mutation** | **H-score value** |
| --- | --- | --- | --- |
| Colon | PIK3CA | H1047R | 0.0145 |
| Ovary | PIK3CA | R88Q | NA |
| Lung | PIK3CA | E545K | 6.03636 |
| Breast | PIK3CA | H1047R | 0.14596 |
| Ovary | PIK3CA | P104L | 7.058 |
| Breast |  | WT | 0.37575 |
| Colon | PIK3CA | I102_P104del | 22.2768 |
| Colon | PIK3CA | E545K | 0.87488 |
| Breast | PIK3CA | E545K | 32.21 |
| Anal canal | PIK3CA | P471A | 156.776 |
| Endometrium | PIK3R1 | H450_E458delinsQ | 20.7635833333333 |
| Endometrium | PIK3CA | E545K | 1.28118 |
| Lung | PIK3CA | E542K | 144.402 |
| Colon | PIK3CA | E545K | 0.723857142857143 |
| Breast | PIK3CA | E542K/E726K | 0.6608 |
| Breast | PIK3CA | H1047L | 1.0453 |
| Breast |  | WT | NA |
| Breast | PIK3CA | E542K | 184.449090909091 |
| Breast |  | WT | 161.775 |
| Breast | PIK3CA | K11E/I112F | 154.777777777778 |
| Liver | PIK3CA | H1047R | 231.877777777778 |
| Breast |  | WT | 0.10868 |
| Breast | PIK3CA | H1047R | 12.15 |
| Breast | PIK3CA | E542K | 173.703333333333 |
| Breast | PIK3CA | H1047R | 8.37325 |
| Endometrium | PIK3CA | I112F | NA |
| Ovary | PIK3CA | E542K | 0.102525 |
| Anal canal | PIK3CA | E542K | 65.2066 |
| Gall bladder | PIK3CA | E542K | 123.755 |
| Breast | PIK3CA | G1049R | 89.62375 |
| Breast | PIK3CA | Q731R/E109del | 2.61346 |
| Breast |  | WT | 0.28948 |
| Breast | PIK3CA | H1047L | 34.26 |
| Cervix | PIK3CA | E542K/E726K | 194.875 |
| Rectum | PIK3CA | E545K | NA |
| Breast |  | WT | 2.106 |
| Cervix | PIK3CA | E542K | 0.32956 |
| Ovary | PIK3CA | E453A | 1.14986666666667 |
| Bladder | PIK3CA | E542Q | 0.39208 |
| Ovary | PIK3CA | E545K | 22.7178 |
| Colon | PIK3CA | A1035V | 4.24566666666667 |
| Breast | PIK3CA | E542K | 21.4294 |
| Stomach | PIK3CA | E545K | 94.925 |
| Breast | PIK3CA | G1049R | 54.1814285714286 |
| Breast | PIK3CA | E542K/G106R | 94.61 |
| Breast | PIK3CA | E542K | 296.176666666667 |
| Breast | PIK3CA | H1047R | 263.552222222222 |
| Breast | PIK3CA | H1047L | 185.496153846154 |
| Breast | PIK3CA | H1047L | 186.411538461538 |
| Breast | PIK3CA | H1047R | 247.5825 |
| Breast |  | WT | 7.0428 |
| Colon | PIK3CA | D926N | 124.903333333333 |
| Endometrium | PIK3R1 | P5688/Q572del | 240.565555555556 |
| Breast | PIK3CA | E545K | 0.29835 |
| Breast | PIK3CA | H1047R | 22.583875 |
| Breast | PIK3CA | H1047L | 1.24718 |
| Breast |  | WT | 1.730175 |
| Breast | PIK3CA | H1047L | 118.158 |
| Breast |  | WT | 0.677791666666667 |
| Breast | PIK3R1 | D569_Q572delinsdel | 87.9754545454545 |
| Breast |  | WT | 0.872733333333333 |
| Breast |  | WT | 0.2437 |
| Anal canal | PIK3R1 | E462_Y463insYDR | 140.471666666667 |
| Breast | PIK3CA | H1047R | 95.715 |
| Breast | PIK3CA | F909L | 259.34375 |
| Breast |  | WT | 0.547766666666667 |
| Colon | PIK3CA | F977L | 0.020625 |
| Breast |  | WT | 6.31944 |
| Larynx | PIK3CA | E81K | 79.1963636363636 |
| Endometrium | PIK3R1 | G376EFSX5/E451_N453delinsD | 185.577142857143 |
| Prostate |  | WT | 297.98 |
| Breast | PIK3CA | E545K | 0.38914 |
| Breast | PIK3CA | H1047R | 180.09875 |
| Breast |  | WT | 1.2926125 |
| Breast | PIK3CA | H1047L | 107.406 |
| Breast |  | WT | 206.73 |
| Anal canal | PIK3CA | E545K | 0.39284 |
| Breast | PIK3CA | E545K | 22.104 |
| Breast | PIK3R1 | c.1746-1_1747delGGT | 279.03 |
| Breast | PIK3CA | H1047L | 75.4775 |
| Pancreas |  | WT | 2.188 |
| Ureter |  | WT | NA |
| Anal canal | PIK3CA | E545K | 30.4923571428571 |
| Breast | PIK3CA | E542K | 0.183588888888889 |
| Breast | PIK3CA | H1047R | 37.8696111111111 |
| Pancreas | PIK3R2 | A2388R244del | 35.6670833333333 |
| Ovary | PIK3R1 | Q455X | NA |
| Colon | PIK3CA | F977L | 0.906 |
| Breast | PIK3CA | E545G | 0.16816 |
| Breast | PIK3CA | H1047R | 60.0505 |
| Breast |  | WT | 0.493416666666667 |
| Breast | PIK3R1 | D464_Y467del/T500I | 10.3888 |
| Breast | PIK3CA | H1047R | 257.69 |
| Breast | PIK3CA | E545K | 57.305 |
| Breast | PIK3CA / PIK3R1 | H1047L / D613del | 1.52057142857143 |
| Breast | PIK3CA | G106_E109del | 294.975 |
| Breast | PIK3CA | E545K | 74.4071428571429 |
| Breast | PIK3CA | H1047R | 56.87 |
| Breast | PIK3CA | E110_N114delinsD | 119.482857142857 |
| Breast |  | WT | 18.47 |
| Breast |  | WT | 5.61725 |
