# Supplementary material for: PIK3CA and PIK3R1 tumor mutational landscape in a pan-cancer patient cohort and its association with pathway activation and treatment efficacy
Source: Sci Rep. 2023 Mar 18;13:4467. doi: 10.1038/s41598-023-31593-w (PMC10024711; doi:10.1038/s41598-023-31593-w)
Supplement: Supplementary file 8 — Supplementary Table 3. [file 41598_2023_31593_MOESM8_ESM.docx]

**Supplemental Table 3 :**

Median value and IQR of H score in each of the tumor groups analysed in figure 5

|  | **H score** | |
| --- | --- | --- |
|  | **Median** | **Inter Quartile Range** |
| **Whole cohort** |  |  |
| PIK3CA Hotspot mutation | 34,26 | 0,9601 - 112,8 |
| PIK3CA Non hotspot mutation | 89,62 | 4,246 - 156,8 |
| PIK3R1 mutation | 140,5 | 20,76 - 240,6 |
| Control (No mutation) | 0,6831 | 0,3061 - 1,859 |
| **Breast tumors** |  |  |
| PIK3CA Hotspot mutation | 66,17 | 14,64 - 115,5 |
| PIK3CA Non hotspot mutation | 89,62 | 27,17 - 224,9 |
| PIK3R1 mutation | No | No |
| Control (No mutation) | 0,6831 | 0,3061 - 1,859 |
| **Gyneco tumors** |  |  |
| PIK3CA Hotspot mutation | 0,8054 | 0,1593 - 17,36 |
| PIK3CA Non hotspot mutation | 7,058 | 7,058 - 7,058 |
| PIK3R1 mutation | No | No |
| Control (No mutation) | 0,6831 | 0,3061 - 1,859 |
| **Digestive tumors** |  |  |
| PIK3CA Hotspot mutation | 37,87 | 0,7994 - 151,9 |
| PIK3CA Non hotspot mutation | 119,5 | 2,698 - 175,8 |
| PIK3R1 mutation | 140,5 | 20,76 - 240,6 |
| Control (No mutation) | 0,6831 | 0,3061 - 1,859 |
