# Supplementary material for: PIK3CA and PIK3R1 tumor mutational landscape in a pan-cancer patient cohort and its association with pathway activation and treatment efficacy
Source: Sci Rep. 2023 Mar 18;13:4467. doi: 10.1038/s41598-023-31593-w (PMC10024711; doi:10.1038/s41598-023-31593-w)
Supplement: Supplementary file 9 — Supplementary Legends. [file 41598_2023_31593_MOESM9_ESM.docx]

**Supplemental Figure 1:** Flow chart of patients studied

**Supplemental Figure 2:**

**A-B:** Prevalence (%) of PIK3CA and PIK3R1 mutations among the major cancer types included in the overall study population (n=1200). White: mutations of PIK3R1; black: hotspot mutations of PIK3CA; gray: non-hotspot mutations of PIK3CA.

**C-D:** Numbers of PIK3CA and PIK3R1 mutations among the major cancer types included in the overall study population (n=1200). White: mutations of PIK3R1; black: hotspot mutations of PIK3CA; gray: non-hotspot mutations of PIK3CA.

**Supplemental Figure 3:**

Objective response rate, Progression-free and Overall survival depending on *PIK3CA* mutational status in the ER + / HER2 - breast cancer population

**A:** Objective response rate to the first line of metastatic chemotherapy

**B**: Progression-free survival to the first line of metastatic chemotherapy

**C:** Overall survival

**Supplemental Figure 4:**

Objective response rate, Progression-free and Overall survival depending on *PIK3CA* mutational status in the triple negative breast cancer population

**A:** Objective response rate to the first line of metastatic chemotherapy

**B**: Progression-free survival to the first line of metastatic chemotherapy

**C:** Overall survival

**Supplemental Figure 5:**

Tumor response and PFS during PI3K-AKT-mTOR inhibition in breast cancer patients

**A:** in the whole cohort of patients with *PIK3CA* mutated tumor

**B**: According to *PIK3CA* mutation type (NHM vs HM)

**C:** According to p-AKT H score
